# Global and regional estimates of dental pain among children and adolescents—systematic review and meta-analysis

**DOI:** 10.1007/s40368-020-00545-7

**Published:** 2020-06-16

**Authors:** Kalyana Chakravarthy Pentapati, Sravan Kumar Yeturu, Hanan Siddiq

**Affiliations:** 1 Department of Public Health Dentistry, Manipal College of Dental Sciences, Manipal, Manipal Academy of Higher Education, Manipal, 576104 Karnataka India; 2 Department of Public Health Dentistry, Amrita School of Dentistry, Amrita Viswavidya Peetham, Kochi, India

**Keywords:** Adolescents, Children, Dental pain, Prevalence, Toothache

## Abstract

**Aim:**

We aimed to evaluate the pooled prevalence of dental pain amongst children and adolescents.

**Methods:**

Studies conducted in children and adolescents up to18 years of age and where prevalence of dental is reported or calculated were included. Search was performed in four major databases from inception to June 1st, 2019. Prevalence estimate at the maximal recall for the dental pain for the individual study was used to calculate the overall pooled estimate.

**Results:**

The prevalence of dental pain ranged from 1.33 to 87.8% in the included publications for quantitative synthesis (*n* = 97). More than half of the publications reported the lifetime prevalence of dental pain (*n* = 51) while few studies reported the current prevalence of dental pain (*n* = 3) and only one study evaluated the dental pain in the past one week. Heterogeneity was high among the included publications (*Q* = 49,063.12; *P* < 0.001; df = 96 and *I*^2^ = 99.8; *P* < 0.001). Overall pooled prevalence of dental pain was 32.7 (CI = 29.6–35.9). No difference was seen with respect to the trends in prevalence of dental pain (Coefficient: 0.005; 95% CI − 0.001–0.011; *P*-value: 0.101).

**Conclusion:**

Two out of ten children below five years, four out of ten children between 6 and 12 years and three out of ten adolescents between 13 and 18 years would have experienced pain in the past. Overall, three out of ten children or adolescents might have experienced dental pain in the past. There was no difference in the pain prevalence between male and females. Studies from Africa reported highest pooled prevalence (50.1%) with least being from Australia (20.7%). Studies from India (40.4%), China (41.3%) and Iran (42.6%) reported high pooled prevalence estimates of dental pain.

## Introduction

Pain is defined as “an unpleasant sensory and emotional experience associated with actual or potential tissue damage, or described in terms of such damage (Treede 2018).” Pain is multidimensional in nature and consists of physiological and psychological variables linked with tissue damage (Santos et al. 2019). Despite considerable improvements in oral health care delivery, dental pain is acknowledged as a common symptom of oral disease (Goes et al. 2008). It is one of the major problems due to which individuals avail dental treatment. It has a considerable impact on the daily activities of individuals like eating, sleeping, homework, paying attention in the class (Goes et al. 2008; Naidoo et al. 2013; Santos et al. 2019), school absenteeism (Ferraz et al. 2014; Ruff et al. 2019), playing, low academic achievement, (Ruff et al. 2019; Seirawan et al. 2012) and avoidance of particular foods which might trigger the pain. Similarly, parents of children having dental pain reported higher workplace absenteeism (Ribeiro et al. 2015b), increased expenditure (2016) and guilt (Gomes et al. 2014). In view of the potential impact of dental pain on oral health-related quality of life, reducing the prevalence of dental pain is included as one of the critical components in the Global Goals for Oral Health 2020 (Hobdell et al. 2003).

Many terminologies like dental pain, oral pain, facial pain, orofacial pain are used interchangeably. Oral pain refers to the pain within the mouth (Macfarlane et al. 2002) whereas dental pain refers to the “pain that originates from innervated tissues within the tooth or immediately adjacent to it (Gibbs and Hargreaves 2013).” “Facial pain (FP) includes pain whose origin is below the canthomeatal line, above the neck and anterior to the ears, while oral pain indicates that the pain is originating from structures within the mouth (Macfarlane et al. 2002).”

Studies have shown a direct relationship of dental pain with oral conditions like dental caries (Moura-Leite et al. 2008), abscess (Ferraz et al. 2014), and dentoalveolar trauma (Moure-Leite et al. 2011). Erupting teeth, as well as exfoliating primary teeth, have also shown to cause dental pain (Shepherd et al. 1999). The most consistent clinical correlate for the occurrence of dental pain is due to the advanced stage of dental caries, primarily in the lower socioeconomic groups with limited access to care (Slade 2001). Young children with dental caries are at risk of experiencing further dental pain (Levine et al. 2002). Even gingivitis has shown to cause dental pain in preschool children (Moura-Leite et al. 2008).

Neurophysiological processes, along with other factors (socio-demographic, cultural, and psychological), have an influential role in the perception of dental pain (Ratnayake and Ekanayake 2005). Adults and children have a different understanding of pain as well as health problems and their effect on oral health-related quality of life (Moura-Leite et al. 2008).

Dental pain was shown to be associated with poor oral health status and decreased access to oral health care which are considered as predictor or proxy indicator to evaluate the use of dental services (Ekanayake and Mendis 2002). Dental pain is a traumatic experience and often requires treatment. Hence, oral health professionals and researchers must understand associated physiological factors, pathological factors, intensity, severity and methods to avoid the dental pain. Considering the impact of dental pain on the individual and the community, it is necessary to understand the scope of this dental public health problem. Such an evaluation helps researchers and planners help to understand the impact of dental pain, frame policies, and the allocation of appropriate and valuable resources (Fernandes et al. 2019). Systematic reviews exist on various clinical oral conditions like caries (Al Agili 2013; Al Ayyan et al. 2018) and molar incisor hypomineralization (Pentapati et al. 2017) in children, dental pain and caries among children and adolescents (Slade 2001), dental pain (Pau et al. 2003) and root caries (Pentapati et al. 2019) in the adults. To date, there were no systematic reviews or meta-analysis on the subjective indicators like the self-reported prevalence of dental pain among children and adolescents. Hence, we aimed to evaluate the pooled prevalence of dental pain amongst children and adolescents.

## Materials and methods

### Selection criteria

Cohort or cross-sectional observational studies which reported period or point prevalence of dental pain among children and adolescents up to18 years of age in English language were included. Studies published as conference proceedings, editorials or letters were excluded.

### Search strategy

Publications were identified by searching four databases (Pubmed, Scopus, Embase, and CINAHL) from inception to June 1st, 2019. Search was performed using keywords and free text words based on the previous publications (“dental pain” OR “teeth pain” OR “tooth pain” OR “toothache” OR “teeth ache” AND “Prevalence” OR “Cross-sectional studies” OR “Epidemiology” OR “Epidemiologic methods” OR “Epidemiologic research design” OR “Epidemiologic studies” OR “Epidemiologic measurements” OR “Cohort studies”) (Mansfield et al. 2016; Pentapati et al. 2019, 2017). Limits applied were children and adolescents up to 18 years of age, humans and English through filters provided by individual databases mentioned above. The studies were transferred to the Rayyan website (https://rayyan.qcri.org/) for the removal of duplicates and screened for titles and then abstracts by evaluators (PKC and YSK). Shortlisted publications were subjected to full-text screening by evaluators (PKC and YSK) to assess the eligibility. Discrepencies were resolved after appraisal by the third evaluator (HS).

### Assessment of risk of bias (ROB)

All publications were appraised for ROB assessment using a nine item tool developed for prevalence studies (Hoy et al. 2012) by evaluators (YSK and HS and discrepancies were resolved by a third evaluator (PKC).

### Data extraction

Two evaluators independently (YSK and PKC) performed the data extraction and any discrepancies were resolved by a third evaluator (HS). Details included were age, gender and geographic distribution, prevalence estimates as per gender, publication year, and prevalence estimates of dental pain (current, 1 week, 1, 3, 6, 12 months, and life-time experience).

### Statistical analysis

Prevalence estimate at the maximal recall for the dental pain for each individual study was used to calculate the overall pooled estimate. *I*^2^ stfatistic which serve as an measure of heterogeneity was calculated. Meta-analysis was performed using Open Meta software (Metafor Package 1.4).(1999) Random-effects model was used to calculate summary prevalence data and 95% confidence intervals (DerSimonian and Laird 1986). Sub-group analysis was performed for variables like continent, gender, risk of bias and time recall of prevalence of dental pain. Meta-regression was performed to evaluate the trends in prevalence of dental pain and Funnel plot was used to assess publication bias (Sterne and Egger 2001).

## Results

### Search results

**Fig. 1:**
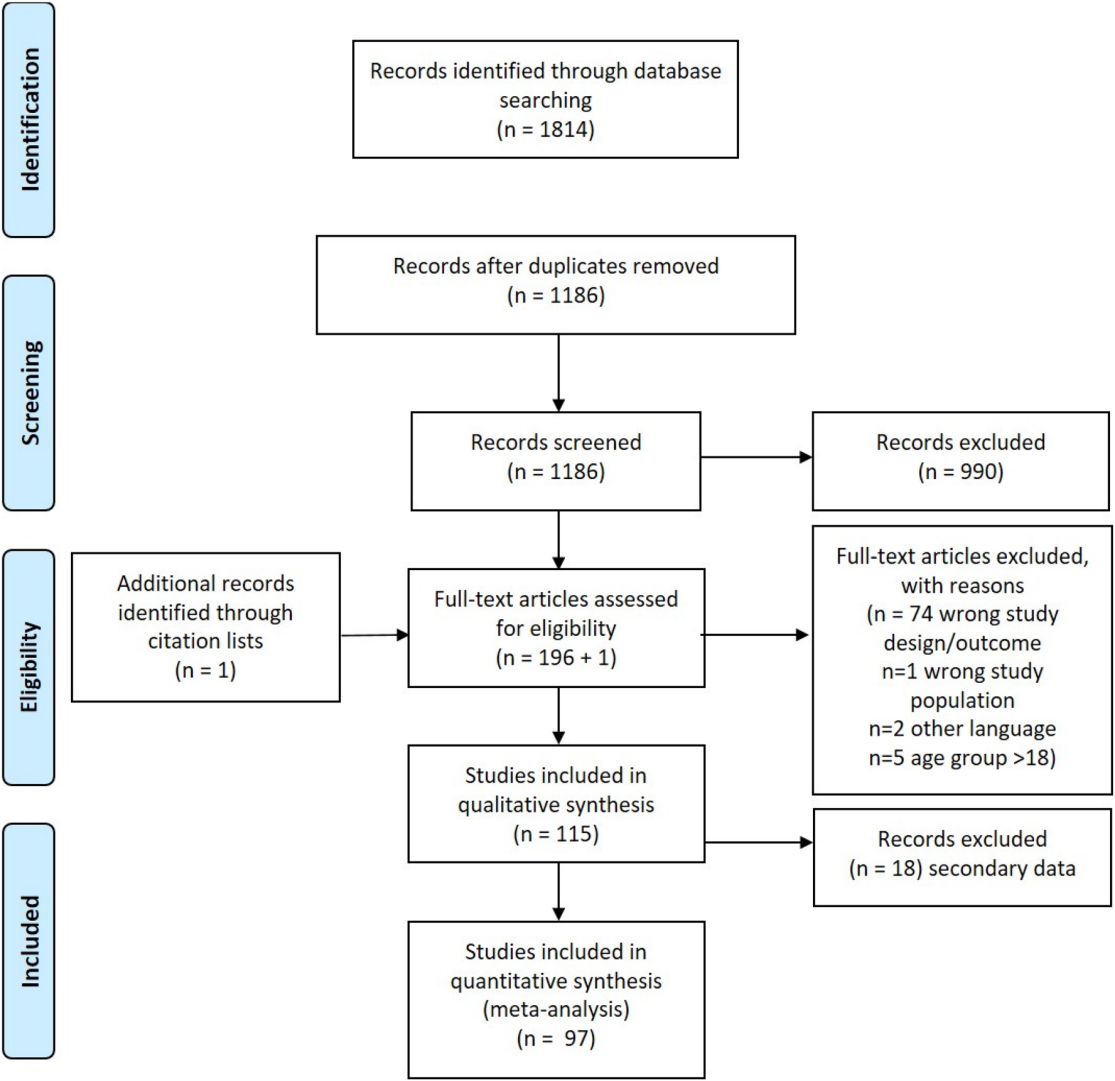
PRISMA flowchart

Our initial search resulted in 1814 publications. After the removal of duplicates, 1186 publications were screened for title and abstract. Full-text screening was performed for eligible publications (*n* = 196). Seventy seven publications were excluded due to inappropriate study design or unclear outcome (*n* = 74), wrong study population (*n* = 1), age of the included participants beyond 18 years (*n* = 5) and other languages (*n* = 2). One publication was included by manual searching of citation list. A total of 115 publications were included in the qualitative synthesis. Eighteen publications were later excluded (secondary data analysis of published studies), and 97 publications were included in the final meta-analysis (Table 1 and Fig. 1).

**Table 1:** Summary characteristics of the included studies

Author and year	Total	Continent	Age	ROB	Prevalence	Questionnaire
(Carmichael et al. 1989)	827	E	5	L	21.77	P
(Treasure and Dever 1992)	342	Au	5	L	16.67	P
(Booth et al. 1992)	227	E	3	L	3.96	P
(Evans et al. 1995)	1185	E	5	L	12.66	P
(Slade et al. 1996)	8568	Au	5–15	L	23.80	P
(Shepherd et al. 1999)	589	E	8	L	47.54	S
(Honkala et al. 2001)	27,765	E	12, 14, 16, 18	L	30.99	S
(Naidoo et al. 2001)	1025	Af	8–10	L	87.80	S
(Ananthakrishnan et al. 2001)	150	As	5–12	L	1.33	U
(Vargas et al. 2002)	560	NA	3–5	L	16.96	P
(De Barrêtto et al. 2004)	601	SA	8–9	L	45.92	S
(Nomura et al. 2004)	169	SA	12–13	L	33.73	P
(Siegal et al. 2004)	2555	NA	2–5	L	9.00	P
(Jiang et al. 2005)	2662	As	11, 13, 15	L	41.28	S
(Kiwanuka and Åstrøm 2005)	614	Af	10–14	L	39.25	S
(Ratnayake and Ekanayake 2005)	576	As	8	L	17.88	S
(Robinson et al. 2005)	174	Af	12	L	36.21	S
(Traebert et al. 2005)	930	SA	12	L	33.98	S
(Vargas et al. 2005)	2411	NA	KG and 3rd grade	L	11.78	P
(Van Dijk et al. 2006)	495	NA	9–13	M	81.62	S
(Bernabé et al. 2007)	805	SA	11–12	L	43.35	S
(Goes et al. 2007)	1052	SA	14–15	L	33.65	S
(Pau et al. 2007)	187	E	12	L	22.46	S
(Shidara et al. 2007)	366	As	6–16	M	68.58	S
(Bastos et al. 2008)	339	SA	12	L	12.09	S
(Moura-Leite et al. 2008)	549	SA	5	L	24.95	P
(Pau et al. 2008)	500	As	11–14	L	30.40	S
(Perera and Ekanayake 2008)	1218	As	15	L	12.32	S
(Campus et al. 2009)	913	E	Secondary school	L	28.26	S
(Jürgensen and Petersen 2009)	594	As	12	L	69.36	S
(Versloot et al. 2009)	652	E	2–5	M	22.09	P
(Jamieson et al. 2010)	301	Au	16–18	L	21.9	S
(Lewis and Stout 2010)	86,730	NA	1–17	L	10.70	P
(Peres et al. 2010)	5815	SA	12 and 15	L	25.54	S
(Rupali et al. 2010)	400	As	14–15	L	7.00	S
(Areai et al. 2011)	9098	Au	9–13	L	22.90	S
(Barrêtto et al. 2011)	174	SA	8–9	L	42.53	S
(Da Silva et al. 2011)	190	SA	11–12	M	45.79	S
(Dandi et al. 2011)	2203	As	12	L	71.40	S
(Dogar et al. 2011)	253	Au	2–4	M	13.44	P
(Du et al. 2011)	14,836	E	3–17	L	10.92	P&S
(Figueiredo et al. 2011)	835	SA	6–7	L	18.92	S
(Jung et al. 2011)	74,689	As	13–18	L	33.90	S
(Jürgensen and Petersen 2011)	612	As	11–13	L	70.26	S
(Ravaghi et al. 2011)	234	As	15–17	M	26.07	S
(Yuen et al. 2011)	153	NA	10–18	M	33.99	S
(Leal et al. 2012)	587	SA	6–7	L	21.81	S
(Ravaghi et al. 2012)	639	As	15–17	L	30.20	S
(Sarri et al. 2012)	965	E	15–16	L	7.36	S
(Tsakos et al. 2012)	292	E	5	L	34.93	S
(Yusof and Jaafar 2012)	132	As	11–12	L	43.94	S
(Colares et al. 2013)	970	SA	5–12	L	44.95	P
(de Lacerda et al. 2013)	385	SA	7–8	L	31.69	S
(Hu et al. 2013)	305	As	7.6–9.3	M	47.54	S
(Lopes et al. 2013)	4249	SA	12	L	23.91	P
(Prasai Dixit et al. 2013)	131	As	8–16	M	31.30	S
(Siqueira et al. 2013)	814	SA	3–5	L	8.72	P
(Kumar et al. 2014)	306	As	10–15	L	34.97	S
(Mulu et al. 2014)	147	Af	6–15	L	27.21	S
(Noro et al. 2014)	688	SA	11–15	L	31.69	S
(Sousa et al. 2014)	732	SA	3–5	L	6.42	P
(Ferreira-Júnior et al. 2015)	7280	SA	5	L	21.94	P
(Karibe et al. 2015)	1415	As	11–15	L	9.96	S
(Lemes et al. 2015)	385	SA	2–4	L	9.87	P
(Ribeiro et al. 2015a)	837	SA	3–5	L	31.66	P
(Schuch et al. 2015b)	750	SA	8–10	L	33.33	S
(Schuch et al. 2015a)	1199	SA	8–12	L	35.70	S
(Soares et al. 2015)	101	SA	6–16	M	57.43	S
(Ul Hasan et al. 2015)	152	As	Primary school	M	42.76	U
(Veiga et al. 2015)	447	E	12–18	M	30.65	S
(Babo Soares et al. 2016)	959	Au	6–17	L	35.77	S
(Nguyen et al. 2016)	556	As	8–10	L	33.45	S
(Shekhawat et al. 2016)	200	As	12–15	L	77.00	S
(Kumar et al. 2016)	800	As	12–15	L	10.13	S
(Adeniyi and Odusanya 2017)	414	Af	8–12	L	61.35	S
(Andegiorgish et al. 2017)	225	Af	12	L	48.00	S
(Barreto et al. 2017)	1367	SA	6–7	L	50.84	S
(Escoffié-Ramirez et al. 2017)	1404	NA	6–12	M	49.86	P
(Ghorbani et al. 2017)	9875	Au	4–17	L	22.79	P
(Guskuma et al. 2017)	1233	SA	12	L	16.71	S
(Kamran et al. 2017)	753	As	4–17	L	10.23	S
(Perazzo et al. 2017)	768	SA	5	L	23.44	P
(So et al. 2017)	1407	NA	6 m–6y	L	37.67	P
(Bashirian et al. 2018)	988	As	7–12	L	71.26	S
(Corrêa-Faria et al. 2018)	563	SA	2–5	L	18.29	P
(Goettems et al. 2018)	1196	SA	8–12	L	16.89	S
(Hardy et al. 2018)	3671	Au	10–16	L	4.09	S
(Hu et al. 2018)	4815	As	12–14	L	52.88	S
(Mishra et al. 2018)	210	As	5–15	M	81.43	S and P
(Misrohmasari et al. 2018)	2377	As	12–14	L	13.93	S
(Oliveira et al. 2018)	9727	SA	13–17	L	59.49	S
(Xu et al. 2018)	1425	As	2–6	L	23.58	P
(Alzahrani 2019)	92	As	12–16	M	79.35	P
(Fernandes et al. 2019)	306	SA	1–3	L	40.20	P
(Maharani et al. 2019)	494	As	12–15	L	17.61	S
(Miao et al. 2019)	7022	As	11–18	L	38.49	S
(Santos et al. 2019)	1589	SA	8–10	L	51.54	S

*SA* South America; *NA* North America; *Af* Africa; *As* Asia; *Au* Australia; *L* Low; *M* Moderate; *S* self-reported; *P* parent reported; *U* Unclear

### Prevalence

The prevalence of dental pain ranged from 1.33 to 87.8% (Ananthakrishnan et al. 2001; Naidoo et al. 2001). Sixteen publications reported a prevalence of more than 50% (Adeniyi and Odusanya 2017; Alzahrani 2019; Barreto et al. 2017; Bashirian et al. 2018; Dandi et al. 2011; Van Dijk et al. 2006; Hu et al. 2018; Jürgensen and Petersen 2011, 2009; Mishra et al. 2018; Naidoo et al. 2001; Oliveira et al. 2018; Santos et al. 2019; Shekhawat et al. 2016; Shidara et al. 2007; Soares et al. 2015). Only ten studies reported a prevalence of less than 10% (Ananthakrishnan et al. 2001; Booth et al. 1992; Hardy et al. 2018; Karibe et al. 2015; Lemes et al. 2015; Rupali et al. 2010; Sarri et al. 2012; Siegal et al. 2004; Siqueira et al. 2013; Sousa et al. 2014) and 18 publications reported prevalence between 10 and 20% (Bastos et al. 2008; Corrêa-Faria et al. 2018; Dogar et al. 2011; Du et al. 2011; Evans et al. 1995; Figueiredo et al. 2011; Goettems et al. 2018; Guskuma et al. 2017; Kamran et al. 2017; Kumar et al. 2014; Lewis and Stout 2010; Maharani et al. 2019; Misrohmasari et al. 2018; Perera and Ekanayake 2008; Ratnayake and Ekanayake 2005; Treasure and Dever 1992; Vargas et al. 2005, 2002). There was high variability among the studies in the assessment of the prevalence of dental pain. The majority of the publications reported the lifetime prevalence of dental pain (*n* = 51), very few studies assessed the current prevalence of dental pain (*n* = 3) and only one study evaluated the dental pain in the past 1 week.

### Age

There was no clear distinction in the age grouping of the children and adolescents for most of the publications. Eighteen publications reported prevalence of pain for children less than 5 years [18.5% (95% CI 14.7–22.4)], 25 publications reported for 6–12 [41.7% (95% CI 33.3–50.2)] years old and only nine publications reported for 13–18 years old [25.8% (95% CI 14–37.5)]. Six studies reported prevalence among WHO index age group 5 years (Range: 12.66–34.93) [21.5% (95% CI 17.1–26)] (Carmichael et al. 1989; Evans et al. 1995; Ferreira-Júnior et al. 2015; Moura-Leite et al. 2008; Perazzo et al. 2017; Tsakos et al. 2012). Eight studies used the WHO index age groups 12 years (Range 12.09–71.4) [30.9% (95% CI 14.1–47.8)] (Andegiorgish et al. 2017; Bastos et al. 2008; Dandi et al. 2011; Guskuma et al. 2017; Jürgensen and Petersen 2009; Lopes et al. 2013; Robinson et al. 2005; Traebert et al. 2005), four studies reported prevalence among 12 and 15 years old (Kumar et al. 2016; Maharani et al. 2019; Peres et al. 2010; Shekhawat et al. 2016) [28.8% (95% CI 12.9–44.7)] and one study reported prevalence in 15 years old (Perera and Ekanayake 2008).

### Gender

Only a quarter of the included publications (*n* = 27) reported gender-specific prevalence of dental pain. The prevalence estimated among males [34.2% (95% CI 29.1–39.2)] was marginally less than females [34.6% (95% CI 28–41.2)].

### Geographic location

Only few studies were reported from Africa (*n* = 6), North America (*n* = 8), and Australia (*n* = 8) continents. However, studies from Africa had highest pooled prevalence of dental pain (50.1%). The majority of the studies were from South America (pooled prevalence = 31%) and Asia (pooled prevalence = 38.6%) (Table 2). Countries with publication of more than three were evaluated for pooled prevalence. Majority of the studies were from Brazil [30.7% (*n* = 31; CI 24.7–36.7)] followed by India [40.4% (*n* = 7; CI 14–66.8)], Australia [21% (*n* = 6; CI 11.2–30.8)], England [18.5% (*n* = 5; CI 7.9–29.2)], USA [21.8% (*n* = 5; CI 14.1–29.5)], China [41.3% (*n* = 4; CI 27.6–55)], Pakistan [27.5% (*n* = 3; CI: 9.2–45.9)], and Iran [42.6% (*n* = 3; CI 11.4–73.7)].

**Table 2:** Subgroup analysis of the pooled estimates (Age, Continent, risk of bias, and dental pain recall)

Category	Number of publications	Estimate (confidence interval)
Age		
≤ 5 years	18	18.5(14.7–22.4)
6–12 years	25	41.7(33.3–50.2)
13–18 years	9	25.8(14–37.5)
Continent		
Europe	12	22.7 (15.3–30.1)
Australia	8	20.7 (13.1–28.3)
Africa	6	50.1 (27–73.1)
Asia	31	38.6 (32.3–44.8)
North America	8	31.3 (21.2–41.5)
South America	32	31 (25.2–36.9)
Risk of Bias		
Low	82	30.1 (26.7–33.5)
Moderate	15	47.4 (35.3–59.5)
Prevalence of dental pain as per the recall		
Current	3	25.1 (9.8–40.5)
1 week	1	20.9
1 month	15	28 (16.3–39.7)
3 months	7	30.7 (18.2–43.3)
6 months	12	27.6 (22.1–33.2)
1 year	17	41.9 (32.6- 51.3)
Life time prevalence	51	31.4 (26.6–36.1)

### Risk of bias

The majority of the publications were in low-risk category (*n* = 82) while very few publications were at moderate risk (*n* = 15). The prevalence estimates for low and moderate ROB publications were 30.1 and 47.4% respectively (Table 2).

### Meta-analysis

**Fig. 2:**
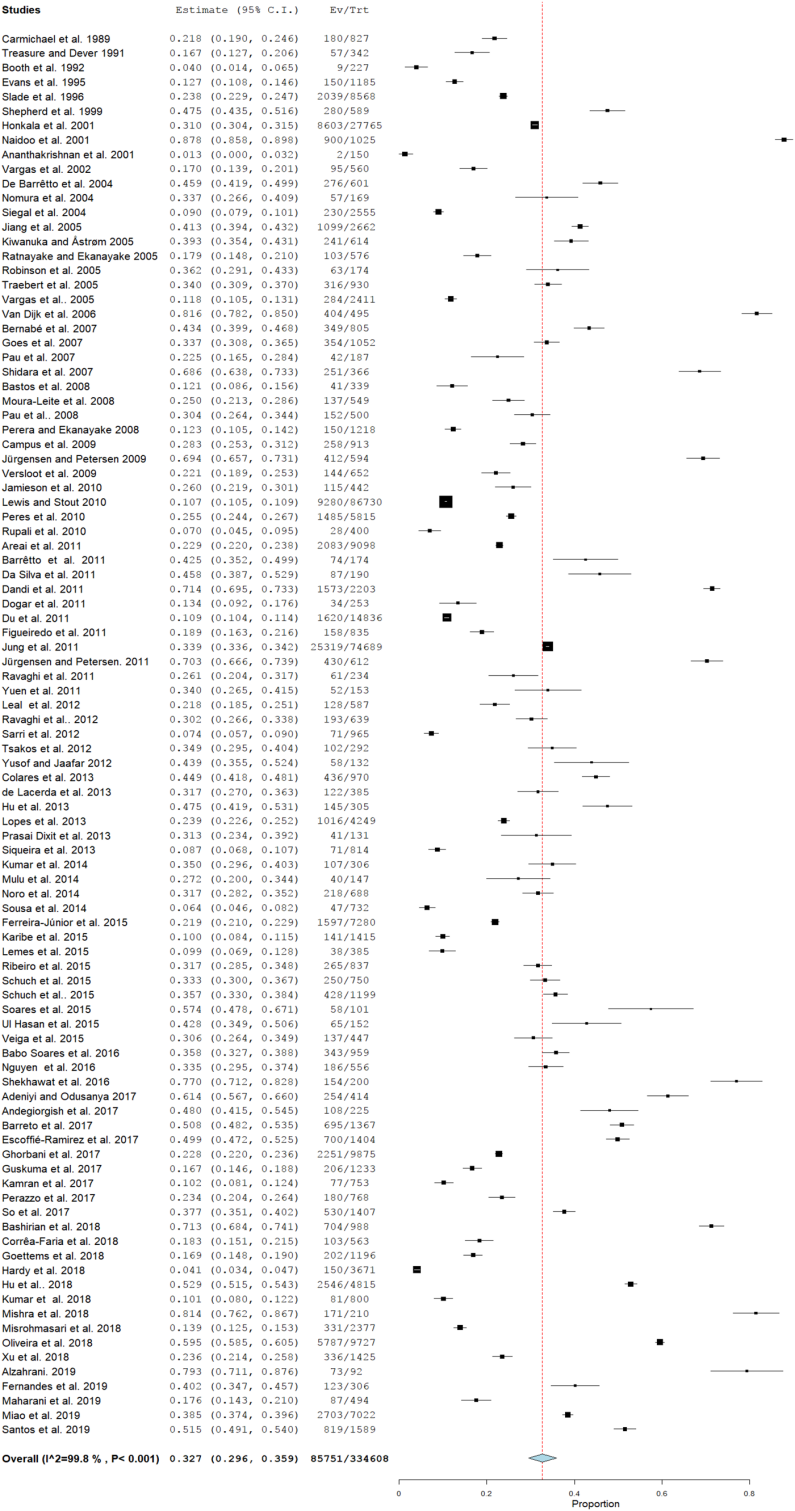
Forest plot of the prevalence of dental pain

**Fig. 3:**
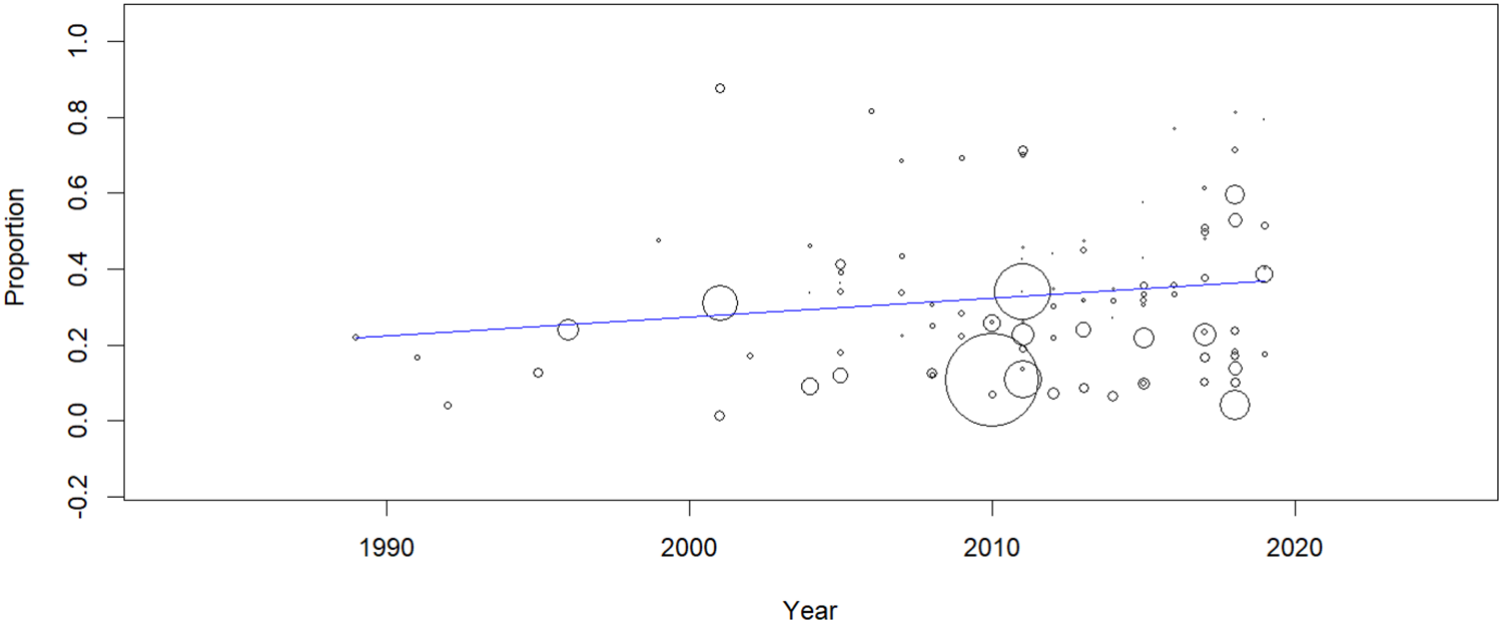
Meta-regression to evaluate the trends of dental pain prevalence

Heterogeneity was high among the included publications (*Q* = 49,063.12; *P* < 0.001; df = 96 and *I*^2^ = 99.8; *P* < 0.001. The random-effects model yielded a pooled prevalence of 32.7 (CI 29.6–35.9) (Fig. 2). Prevalence of dental pain in publications was reported as current, 1 week, 1 month, 3, 6, 12 months and a lifetime experience. Accordingly, the pooled prevalence of dental pain was presented in Table 2. No difference was seen in the trend of dental pain over the years (Coefficient: 0.005; 95% CI − 0.001–0.011; *P*-value: 0.101). (Fig. 3).

### Publication bias

**Fig. 4:**
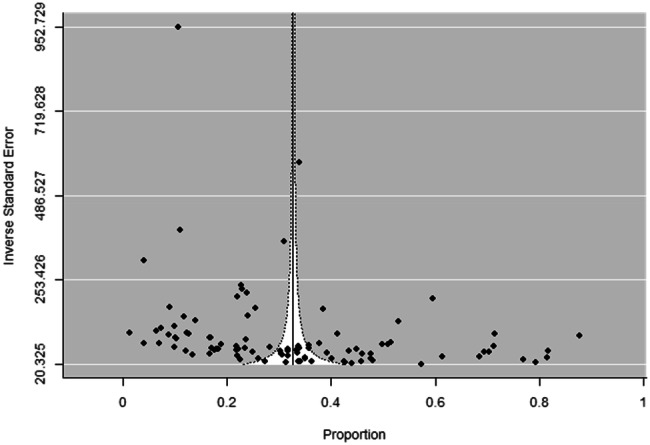
Publication bias in prevalence estimates of dental pain with inverse standard error

The funnel plot showed asymmetry (*p* < 0.001) (Fig. 4).

## Discussion

Dental pain can be a preventable and or treatable condition, although it may be self-limiting in few cases. In this review, we aimed to evaluate the pooled prevalence of dental pain among children and adolescents through subjective self/proxy reports of dental pain in children and adolescents. A total of 97 studies constituted for the pooled estimates in this meta-analysis. The pooled prevalence has to be interpreted with caution due to high heterogeneity among the included publications. High heterogeneity could be due to the variability in disease prevalence which could have caused the dental pain, recall time interval for the prevalence of dental pain, geographic variation, access to care, social status, and availability of services. The overall pooled prevalence of dental pain was 32.7%. Large population or national surveys reported prevalence in the range of 10.7–59.5% (Ferreira-Júnior et al. 2015; Jung et al. 2011; Lewis and Stout 2010; Lopes et al. 2013; Misrohmasari et al. 2018; Oliveira et al. 2018; Peres et al. 2010). Studies from Africa reported high pooled prevalence (50.1%) and lowest pooled prevalence was seen in Australia (20.7%). Females showed marginally higher prevalence estimates than males. There was no significant difference in the trend of dental pain prevalence over the three decades. Among the included studies, age and gender-specific prevalence estimates have not been reported adequately.

There was no consensus in the recording of dental pain among the included studies. Studies have used single-item questions or questionnaires to evaluate the history of dental pain. Also, there was diversity in the recall time interval used across the studies viz., current, 1 week, 1 month, 3, 6, 12, and a lifetime experience of dental pain. Few studies used more than one recall time interval which creates ambiguity in prevalence estimates. Nevertheless, we have used the maximal recall time interval from each study to calculate the overall prevalence of dental pain.

Dental pain could be due to a variety of reasons (eruption, exfoliation, carious teeth, dentinal hypersensitivity, or abscess) among children adolescents. The reason for the dental pain in most of the studies was not emphasized. Most of the reasons which cause dental pain may need treatment from a dental professional while reasons like eruption and exfoliation could be self-limiting. There could be overall inflation of the prevalence estimates due to the lack of emphasis on the etiology of the dental pain in the published studies.

Exclusion of non-English studies, lack of age specific prevalence estimates for substantial number of publications, and reason for dental pain are few of the limitations. Within the limits of this review, we could conclude that three out of ten children or adolescents could have experienced dental pain in the past. However, the prevalence estimates of dental pain may not indicate the exact treatment need due to reasons like self-limiting pain. It can be indicative of the burden of disease and its impact on children and adolescents. Such data may be useful for planning public health programs. Dental professionals should consider the fact that dental pain can be a common symptom among children and adolescents and is based on the subjective feeling of the individual.

Future studies on the prevalence of dental pain should use the the standard guidelines of “Strengthening the Reporting of Observational Studies in Epidemiology (STROBE)” and widely accepted criteria for the recall time interval for self-reported dental pain. Emphasis should be made on the reasons for dental pain to prevent overestimation of prevalence.

## Conclusion

Considering the limitations of the study, two out of ten children below 5 years, four out of ten children between 6 and 12 years and three out of ten adolescents between 13 and 18 years would have experienced pain in the past. Overall, three out of ten children or adolescents might have experienced dental pain in the past. There was no difference in the pain prevalence between male and females. Studies from Africa reported highest pooled prevalence (50.1%) with least being from Australia (20.7%). Studies from India (40.4%), China (41.3%) and Iran (42.6%) reported high pooled prevalence estimates of dental pain.
